# The Octadecanoid Pathway, but Not COI1, Is Required for Nectar Secretion in *Arabidopsis thaliana*

**DOI:** 10.3389/fpls.2018.01060

**Published:** 2018-08-08

**Authors:** Anthony J. Schmitt, Rahul Roy, Peter M. Klinkenberg, Mengyuan Jia, Clay J. Carter

**Affiliations:** Department of Plant and Microbial Biology, University of Minnesota Twin Cities, St. Paul, MN, United States

**Keywords:** nectar, nectaries, jasmonic acid, auxin, F-box proteins, SWEET9

## Abstract

Over 75% of crop species produce nectar and are dependent on pollinators to achieve maximum seed set, yet little is known about the mechanisms regulating nectar secretion. The phytohormone jasmonic acid (JA) is recognized to be involved in several plant processes including development and defense. JA was also recently shown to positively influence nectar secretion in both floral and extrafloral nectaries. For example, endogenous JA levels peak in flowers just prior to nectar secretion, but the details of how JA regulates nectar secretion have yet to be elucidated. We have found that the octadecanoid pathway does indeed play a role in the production and regulation of floral nectar in Arabidopsis. Null alleles for several JA biosynthesis and response genes had significantly reduced amounts of nectar, as well as altered expression of genes known to be involved in nectar production. We additionally identified crosstalk between the JA and auxin response pathways in nectaries. For example, the nectar-less JA synthesis mutant *aos-2* showed no auxin response in nectaries, but both nectar production and the auxin response were restored upon exogenous JA and auxin treatment. Conversely, *coi1-1*, a JA-Ile-insensitive receptor mutant, displayed no auxin response in nectaries under any circumstance, even in older flowers that produced nectar. Surprisingly, *opr3-1*, a mutant for *12-oxophytodienoate reductase* 3 [an enzyme further down the JA biosynthetic pathway that reduces 12-oxo phytodienoic acid (OPDA)], produced no nectar in newly opened flowers, but did secrete nectar in older flowers. Furthermore, a similar phenotype was observed in *coi1-1*. Cumulatively, these observations strongly suggest an indispensable role for an octadecanoic acid- and auxin-dependent, but JA- and COI1-dispensible, pathway in regulating nectar production in Arabidopsis.

## Introduction

Nectar is a major factor for enhancing plant-mutualist interactions. Nearly 90% of all plant species, including 75% of domesticated crops, benefit from animal-mediated pollination, which is usually facilitated by floral nectar (Klein et al., 2007; Calderone, 2012). Floral nectar is offered to increase pollinator visitation, while extra-floral nectar is used to attract mutualist insects that provide protection from herbivory (Heil, 2011). Despite the importance of nectar in attracting pollinators to promote outcrossing and help plants achieve maximum seed set, relatively little is known about the molecular regulation of nectar secretion.

*Arabidopsis thaliana* flowers, although highly self-fertile, surprisingly still produce nectar, which is thought to contribute to outcrossing events in natural populations (Hoffman et al., 2003; Kram and Carter, 2009). Due to the wealth of genomic resources and its close relatedness to agriculturally important relatives in the Brassicaceae family – that often require effective pollinator visitation for achieving maximum seed set – Arabidopsis has been an important model for studying the genetic and molecular mechanisms required for nectary function (Kram and Carter, 2009). Arabidopsis flowers have two types of nectaries: median and lateral. The lateral nectaries are located at the base of the short stamen and secrete >99% of total floral nectar (Davis et al., 1998; Kram and Carter, 2009). Median nectaries are located at the base of petals and long stamens and produce little to no nectar. Immature lateral nectaries accumulate starch, which is then broken down at anthesis and the resulting sugars are eventually secreted into the floral nectar (Stage 13–15; newly opened flowers, pollen shed and nectar secretion) (Ren et al., 2007; Kram and Carter, 2009). The nectar produced by these lateral nectaries of Arabidopsis (and most Brassicaceae species) is hexose-rich (nearly all glucose and fructose) (Davis et al., 1998).

A few recent reports have enhanced our understanding of nectar production. The current literature increasingly supports an eccrine-based secretion model for floral nectar in the Brassicaceae (Roy et al., 2017). Eccrine-based secretion relies on plasma membrane-localized pores and transporters to export nectar metabolites from parenchymal cells in the nectary. In one model, nectary starch is degraded and re-synthesized into sucrose by sucrose phosphate synthases (SPS) and other enzymes (Lin et al., 2014). Next, the sucrose is exported into the apoplastic space via the sucrose uniporter AtSWEET9 (Lin et al., 2014). In the apoplastic space CELL WALL INVERTASE 4 (AtCWINV4) catalyzes the hydrolysis of sucrose into hexose monomers, glucose and fructose (Ruhlmann et al., 2010). This invertase activity creates both a constant driving force for sucrose export and a negative water potential causing water to move toward sugars and create water droplets. Perhaps not surprisingly, knockout mutants of *SPS, SWEET9*, and *CWINV4* all lack nectar production. Although we are beginning to understand key genes involved in the process of nectar secretion, the ways in which these processes are regulated is also still poorly understood.

In order to support effective mutualist visitation and proper pollination, floral nectar production must be carefully coordinated with petal opening, pollen shed, stigma receptivity and pollinator activity. Therefore, it is not surprising that floral nectar production would require hormonal regulation to ensure its production is tightly coordinated with these other important processes. Even though proper regulation of nectar secretion is essential to its overall function – effectively manipulating pollinator visitation (Pyke, 2016) – the impacts of each of the phytohormones has remained rather elusive in relation to nectar production. Some studies have examined the relative impacts of auxin (IAA, indole acetic acid) (Bender et al., 2013), gibberellins (Wiesen et al., 2016), and jasmonic acid (JA) (Radhika et al., 2010) with regard to their roles in regulating floral nectar secretion as outlined below.

It is well established that auxin is an important phytohormone that heavily regulates both developmental processes as well as responses to biotic and abiotic stresses (Chapman and Estelle, 2009; Lokerse and Weijers, 2009; Leyser, 2010; Zhao, 2010; Weijers et al., 2018). IAA activates transcriptional responses through binding to the TIR1 F-box receptor, which leads to the ubiquitin-mediated degradation of AUX/IAA transcriptional repressors and the de-repression of auxin response factors (ARFs) thus activating auxin response genes (Dharmasiri et al., 2005). IAA is important for regulating proper floral development too (Aloni et al., 2006). Furthermore, recent reports have shown that auxin-related genes display nectary-enriched expression profiles in the Brassicaceae (Kram et al., 2009; Hampton et al., 2010). Interestingly, *PIN6*, an auxin efflux transporter, was shown to have a positive effect on total nectar production as *PIN6* overexpressers showed an increase in total nectar production and *PIN6* knockdown mutants show a decrease in nectar production in Stage 14–15 flowers (Bender et al., 2013). When Arabidopsis inflorescences were treated with exogenous auxin there was a 2 – 10-fold increase in total nectar production in Arabidopsis and *Brassica napus*, whereas the auxin transport inhibitor NPA decreased nectar output (Bender et al., 2013).

In addition to IAA, JA is another phytohormone known to play important roles in plant flower development as well as plant defense (Wasternack and Hause, 2013). JA is a lipid-derived hormone whose biosynthesis occurs via the octadecanoic pathway and begins with the cleaving of alpha-linolenic acid (C18:3) off of lipid bilayers by phospholipases (e.g., Ishiguro et al., 2001) (see **Supplementary Figure S1** for diagram of full synthetic pathway). The primary mode of JA signaling occurs through a similar mechanism as described for IAA. JA signals through the COI1 F-box protein that forms a Skp-Cullin-F-box (SCF) ubiquitin ligase complex that leads to the ubiquitination and degradation of JAZ repressors in the presence of jasmonoyl-L-isoleucine (JA-Ile), the bioactive form of JA (Kelley and Estelle, 2012; Nagels Durand et al., 2016). Some previous reports have shown that JA plays a role in the regulation of nectar production. For example, JA levels in *B. napus* flowers peak just prior to anthesis which, as previously mentioned, is coincidental with the onset of nectar production (Radhika et al., 2010). Furthermore, exogenous floral application of phenidone, a chemical inhibitor of JA synthesis, lowered nectar production while exogenous JA increased total nectar (Radhika et al., 2010). In tobacco, JA’s importance in regulating nectar secretion is also evident. Tobacco flowers silenced for JA synthesis and response have no nectar and show altered starch utilization (Liu and Thornburg, 2012; Wang et al., 2014). For example, the JA-responsive transcription factor NtMYB305 was shown to be required for nectary maturation and nectar secretion (Liu et al., 2009; Liu and Thornburg, 2012; Wang et al., 2014). These mutants also showed defects in starch accumulation. Furthermore, Stitz et al. (2014) showed that JA-Ile signaling through COI1 is required for nectar production in tobacco flowers. Although there is a small body of work implicating that JA and auxin are involved in nectary maturation and nectar secretion, the details as to exactly how these hormones regulate these processes is still an area that requires further investigation.

Phytohormones do not work in isolation and the coordinated interactions between JA, IAA, and GA make it difficult to study the sole influence that each of these hormones may have on nectary function. For instance, in Arabidopsis IAA acts through ARF6 and ARF8 to induce JA synthesis leading to the expression of *MYB21* and *MYB24* which together play important roles in flower maturation (Nagpal et al., 2005; Reeves et al., 2012). To add to the complexity of this hormonal coordination, gibberellin-deficient flowers show reduced levels of JA and lower expression of *MYB21/2*4 (Cheng et al., 2009). Using combinations of JA biosynthesis and response mutants, in addition to transgenic lines with altered auxin levels, here we demonstrate that the crosstalk between JA and IAA is essential for the regulation of nectary function.

## Materials and Methods

### Plant Material and Growth Condition

All plants were grown on peat-based medium with vermiculite and perlite (Pro-Mix BX; Premier Horticulture) in individual pots. Arabidopsis seeds were sterilized in a 33% bleach solution + 0.01% Triton mix for 10 min and subsequently washed five times with water before being planted. The plants were either housed in a Percival AR66LX growth chamber with 16 h day/8 h night, photosynthetic flux of 150 μmol m^-2^ sec^-1^ and at 23°C or in a growth room with same 16 h day/8 h night cycle at 22°C. It should be noted that plants used for any direct comparisons were grown at the same time, in the same trays, under the same growth conditions to limit environmental effects on nectar production. Arabidopsis seed for wild-type (Col-0), *dad1-1* (SALK_138439), and *jar1-11* (CS67935) were obtained from the Arabidopsis Biological Resource Center (ABRC). DR5::GUS plants were previously described (Ulmasov et al., 1997). Homozygous mutants of *myb21-4, opr3-1*, *coi1-1*, and *aos-2* (SALK_017756) were provided by the Reed Group at the University of North Carolina at Chapel Hill (Reeves et al., 2012). Both *coi1-1* and *myb21-4* are sterile and not rescuable via exogenous JA application, as such they were carried as heterozygous mutants by backcrossing to wild-type Col-0 and subsequent selection for homozygous mutants in downstream experiments. Jas9-VENUS (Stock N2105629) and mJas-9 Venus (Stock N2105630) seeds were ordered from Nottingham Arabidopsis Stock Center (NASC) and previously described by Larrieu et al., 2015. All T-DNA mutants were genotyped with gene specific primers (**Supplementary Table S1**) flanking the T-DNA insertion site and the T- DNA specific primer “LBb1.3” to screen for the T-DNA insert as described at: http://signal.salk.edu/tdnaprimers.2.html.

Plants expressing the auxin synthesis gene *iaa*M under the control of the nectary-specific SWEET9 promoter were generated by PCR amplifying the *iaa*M gene out of *Pseudomonas savastanoi* genomic DNA using the primer pair “*iaaM* ORF-F” and “*iaaM* ORF-R” and then ligating it into the *Xma*I and *Sme*I sites downstream of the nectary-specific SWEET9 promoter in the plant transformation vector pPMK1 (Bender et al., 2012). This vector was subsequently given the name pPMK21. *Agrobacterium tumefaciens* (GV3101) cells were transformed to carry the pPMK21 vector and used to transform Arabidopsis using the floral-dip method described by Clough and Bent (1998). Transformed seedlings were selected on one half Muarshige and Skoog medium plates with 50 μg/ml kanamycin.

### Hormone Treatment of Flowers

1-naphthalene acetic acid (1-NAA) (Cat #N0640) and methyl jasmonate (Cat #392707) were obtained from Sigma-Aldrich. 1-NAA stocks were prepared in ethanol. Treatment of flowers with 10 μM 1-NAA or 500 μM methyl jasmonate was conducted by dipping the inflorescence tips in the appropriate hormone solution dissolved in a 0.05% aqueous Tween 20 solution the evening before testing nectar production. Flowers were phenotyped for the production of nectar and anther dehiscence, or RNA was isolated via the protocol described below.

### Gene Expression Analysis

For qRT PCR analysis, RNA was isolated from tissues (minimum of 10 flowers per biological replicate) with the Absolutely RNA Miniprep kit according to the manufacturer’s instructions (Agilent, Catalog #400800). RNA quality was confirmed by spectrophotometric analysis and agarose gel electrophoresis. For cDNA preparation, total RNA (500–1,000 ng) was used as template for Promega’s Reverse Transcription System (Promega, Catalog #A3500). 10 ng of the resulting cDNA was added to the real time PCR reaction mix, which included 10 μl of 2× Brilliant III Ultra-Fast QPCR Master Mix (Agilent Technologies, Cat #600880), 0.4 μl of each forward and reverse primer (10 μM stock), 0.4 μl ROX dye (high), and 6.8 μl nuclease-free H2O. Primers were designed using the online primer design tool “QuantPrime.” The Applied Biosystems StepOnePlus thermocycler was used for real-time PCR, and results were analyzed with Applied Biosystems StepOne software (v2.3). Three biological replicates with a minimum of two technical replicates were performed for each experiment.

### Histochemical Staining for Starch and GUS

Starch staining was performed as described by Ruhlmann et al. (2010). Freshly collected flowers were dipped in Lugols iodine solution (Fischer, Cat # S93408) for 5 min after a 1-min vacuum infiltration in the same. Sepals were removed carefully before beginning staining to improve stain permeation. Flowers were subsequently rinsed in deionized water twice to wash off excess stain and subsequently imaged under a dissecting microscope. GUS staining was performed as described previously (Jefferson et al., 1987). For GUS staining, flowers were dissected to remove sepals and subsequently dipped in a GUS staining solution, kept under 15 psi vacuum for 15 min and incubated at 37°C for 16 h. The stain was subsequently removed and the flowers were washed with 90% ethanol thrice, with each wash lasting 2–3 h. The final wash was with 70% ethanol overnight. The flowers were then transferred to deionized water and imaged under a dissecting microscope.

### Microscopic Analysis

Flowers expressing mJas9-Venus or Jas9-Venus were viewed either using an Olympus BX-53 fluorescence microscope (YFP cube) or a Nikon A1si spectral confocal microscope attached to a Nikon TE2000 motorized inverted microscope (Nikon USA, Melville, NY, United States) using the 514 nm laser line (laser power 25.4). The emitted fluorescence signal was collected using the 32-channel spectral detector at 6.0 nm spectral resolution using the 20× objective (n.a. 0.75) with zoom setting of 4. Optical sections were collected at 0.5 μm increments then spectrally unmixed using Nikon Elements software ver. 5.1.2.

## Results

### Exogenous Methyl Jasmonate Induces Nectar Secretion in Arabidopsis JA Synthesis Mutants

**FIGURE 1:**
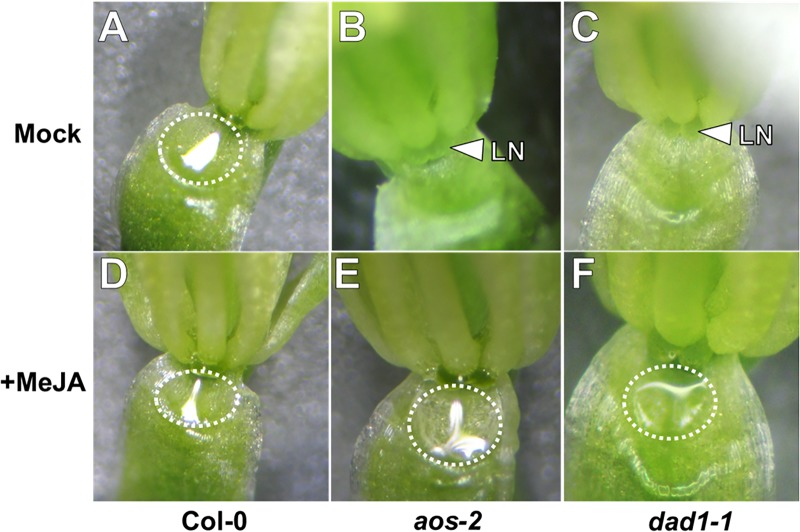
Exogenous methyl jasmonate induces nectar secretion in Arabidopsis JA synthesis mutants. Mock treated wild-type flowers at Stage 14 (fully open) secrete nectar **(A)**, whereas the equivalent flowers of JA synthesis mutants (*aos-2*, **B** and *dad1-1*, **C**) do not. Exogenous application of MeJA (500 μM; **D–F**) induces nectar secretion in JA synthesis mutant flowers **(E,F)**. Arrowheads indicate the location of the lateral nectaries (LN) and dashed circles outline the presence of nectar droplets.

Jasmonic acid has been suggested to play a role in nectar production (Heil, 2001; Radhika et al., 2010; Wang et al., 2014). In order to examine the role of JA in Arabidopsis nectar production, we first observed mutants of the JA biosynthesis genes *DELAYED ANTHER DEHISCENCE* (*DAD1*, AT2G44810) and *ALLENE OXIDE SYNTHASE* (*AOS*, AT5G42650). *AOS* is a single copy gene indispensable for JA synthesis (Park et al., 2002), whereas *DAD1* encodes a filament-specific phospholipase involved in JA synthesis in flowers, but plants are not entirely JA deficient (Ishiguro et al., 2001). The first two open flowers (equivalent of Stage 14 in Col-0) of both *aos-2* and *dad1-1* produced no nectar while appearing to maintain proper nectary morphology (**Figures 1B,C**). The mutants also exhibited male sterility (data not shown), which was a previously known phenotype to each mutant (Ishiguro et al., 2001; Park et al., 2002). When these synthesis mutants were exogenously treated with the volatile methylated form of JA, methyl jasmonate (MeJA), nectar production was rescued in recent fully opened flowers (equivalent to Stage 14 flowers in wild-type; **Figures 1E,F**). Wild-type plants showed no obvious change in nectar volume in response to MeJA treatment (**Figure 1D**). Since JA synthesis mutants are sterile, their flowers do not quickly dehisce like wild-type, instead their ‘older’ flowers (third open flower and down from the meristem) remain open. Interestingly, these ‘older’ open flowers in *dad1-1* produced nectar, whereas those of *aos-2* did not (**Supplementary Figures S1, S2**). The relationship between nectar secretion in ‘young’ and ‘old’ flowers of JA synthesis and response mutants is further explored below. Cumulatively, these results further support an essential role for JA in regulating nectar production.

### Exogenous Methyl Jasmonate and Auxin Induce Nectar Secretion and Auxin Responses in *aos-2* Nectaries

**FIGURE 2:**
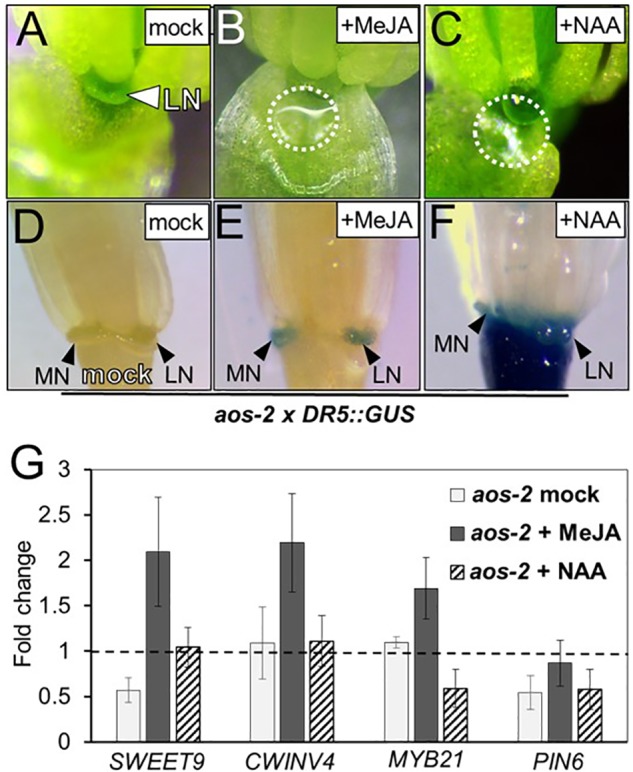
Exogenous methyl jasmonate and auxin induce nectar secretion and auxin responses in *aos-2* nectaries. Mock treated *aos-2* flowers do not secrete nectar **(A)** and lack the nectary auxin response **(D)** observed in wild-type plants. Treatment of *aos-2* flowers with either MeJA (500 μM) or the synthetic auxin NAA (10 μM) induces nectar secretion **(B,C)** and the nectary auxin response **(E,F)**. The expression of key genes involved in nectar production were evaluated in whole *aos-2* Stage 14 flowers after treatment with MeJA or NAA by qRT-PCR **(G)**. Data is presented as fold-change in expression of each gene normalized to Col-0 (dashed line).

Bender et al. (2013) previously showed that auxin homeostasis is important for nectar secretion. Cross talk between auxin and JA is also important for floral development (Nagpal et al., 2005; Varaud et al., 2011; Reeves et al., 2012), therefore we hypothesized that JA may be involved in crosstalk with auxin to regulate nectary function and an altered auxin response would be observed in the JA mutants. To further explore this hypothesis, *aos-2* was crossed into the auxin-responsive DR5::GUS reporter line and whole flowers were subjected to histochemical GUS staining (Ulmasov et al., 1997). In the wild-type background the DR5::GUS reporter showed a strong auxin response in both the median and lateral nectaries (**Supplementary Figure S2A**). However, there was no signal in the nectaries of the *aos-2* background, which produce no nectar (**Figures 2A,D**). When flowers were treated with MeJA, both nectar production and the nectary auxin response were rescued (**Figures 2B,E**).

Since previous reports have shown auxin treatment can increase nectar production (Bender et al., 2013), we decided to test if exogenous application of 1-naphthaleneacetic acid (NAA) – a synthetic membrane permeable auxin – could rescue nectar production in JA mutants. As with MeJA, floral crowns exogenously treated with 10 μM NAA displayed a restoration of nectar production (**Figure 2C**).

Given the dramatic change of nectar production in these JA biosynthesis mutants, we examined the expression of previously characterized genes known to be involved in nectar production in *aos-2* flowers exogenously treated with either MeJA or NAA (**Figure 2G**). Genes targeted for qRT-PCR analysis in JA mutants included *SWEET9*, a sucrose transporter (Lin et al., 2014), *CWINV4*, an invertase responsible for cleaving sucrose into hexose sugars (Ruhlmann et al., 2010), *PIN6*, an auxin efflux transporter (Bender et al., 2013) and *MYB21*, an ortholog of the JA-inducible tobacco transcription factor MYB305, which is required for nectar secretion (Liu et al., 2009; Liu and Thornburg, 2012; Reeves et al., 2012; Wang et al., 2014).

*SWEET9* and *PIN6* transcripts were downregulated in nectar-less mock treated *aos-2* flowers relative to Col-0 (wild-type) (**Figure 2G**), which in itself could explain the loss of nectar production (Bender et al., 2013; Lin et al., 2014). Exogenous treatment of MeJA restored nectar production (**Figure 2E**) and induced the expression of *SWEET9*, *CWINV4*, and *MYB21* by ∼2-fold compared to wild-type and ∼4-fold relative to the mock treated *aos-2* specifically for *SWEET9* (**Figure 2G**). *aos-2* flowers treated with exogenous NAA also produced nectar and restored *SWEET9* transcript to wild-type levels, but *MYB21* transcript abundance was downregulated and had little effect on *PIN6* transcript level relative to mock-treated Col-0 (**Figure 2G**).

### Nectary-Derived Auxin Can Rescue Nectar Secretion in *aos-2*

**FIGURE 3:**
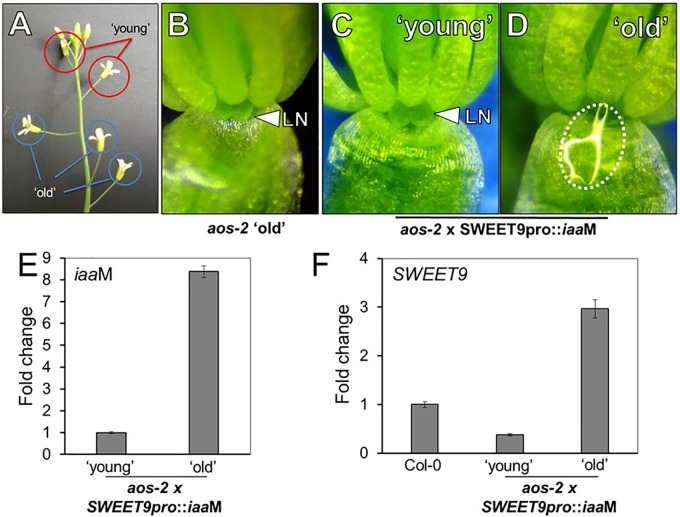
Endogenous auxin induces nectar secretion in *aos-2*. **(A)** Relative locations of ‘young’ and ‘old’ flowers in JA-synthesis mutants, like *aos-2*. ‘Old’ *aos-2* flowers produced either no nectar **(B)** or a very faint glistening of fluid on the inner surface of the sepal. ‘Young’ *aos-2* flowers expressing the auxin synthesis gene *iaa*M under control of the SWEET9 promoter do not produce nectar **(C)**, whereas the corresponding ‘old’ flowers produce large nectar droplets **(D)**. **(E)** *iaa*M expression is strongly induced in ‘old’ *aos-2* × SWEET9pro::*iaa*M flowers (qRT-PCR data normalized to ‘young’ flowers). **(F)** Expression of *SWEET9* transcripts in Col-0 and ‘young’ and ‘old’ *aos-2* × SWEET9pro::*iaa*M flowers (qRT-PCR data normalized to Col-0 flowers).

Exogenous auxin (NAA) restored nectar production in *aos-2* flowers (**Figure 2**), but since NAA could only be applied to whole flowers it was not clear if the auxin-dependent signaling was limited to the nectaries. Thus, we decided to engineer transgenic *aos*-2 that could produce high endogenous auxin specifically in the nectaries by expressing the auxin biosynthesis gene *iaa*M (Klee et al., 1987) under control of the nectary-specific SWEET9 promoter (Bender et al., 2013; Lin et al., 2014). This construct, SWEET9pro::*iaa*M, was first transformed into wild-type plants. The subsequent SWEET9pro::*iaa*M transgenic line was then crossed into *aos-2* and phenotyped. The first two fully open flowers (‘young,’ **Figure 3A**) of *aos-2* x SWEET9pro::*iaa*M did not produce nectar (**Figure 3C**) similar to both the ‘young’ and ‘old’ flowers of *aos-2* alone (**Figures 1B**, **3B**). The ‘old’ flowers (open flowers 3–5 of down from the meristem) of *aos-2* × SWEET9pro::*iaa*M did produce nectar (**Figure 3D**). Hence the nectary-derived auxin eventually caused nectar to be produced in the *aos-2* background. It should be noted that the anthers never dehisced in *aos-2*, either with or without SWEET9pro::*iaa*M, so fertilization was not a cause of the auxin signaling.

To determine if gene expression could account for nectar secretion observed in *aos-2* × SWEET9pro::*iaa*M, we first verified that *iaa*M expression was induced in ‘old’ vs. ‘young’ flowers (**Figure 3E**). We hypothesized that the low expression of *SWEET9* in the *aos-2* background (**Figure 2G**) would correspondingly keep *iaa*M levels low in younger *aos-2* × SWEET9pro::*iaa*M flowers. The gradual increase in endogenous auxin, driven by control of the SWEET9 promoter, subsequently would result in a positive feedback loop and nectar production in ‘old’ *aos-2* × SWEET9pro::*iaa*M flowers. Indeed, *SWEET9* transcripts in *aos-2* × SWEET9pro::*iaa*M flowers were significantly downregulated in ‘young’ flowers, but upregulated >3-fold in ‘old’ flowers relative to Stage 14 Col-0 flowers (open and secreting nectar) (**Figure 3F**). As a control, flowers from both ‘young’ and ‘old’ *aos-2* not harboring the SWEET9pro::*iaa*M transgene were examined for *SWEET9* expression, which demonstrated that SWEET9 is not induced in ‘old’ flowers not carrying *iaa*M (**Supplementary Figure S3**), suggesting that SWEET9 expression in *aos-2* × SWEET9pro::*iaa*M flowers is dependent on endogenous auxin synthesis. These results cumulatively support the hypothesis that endogenous auxin can rescue *aos-2* nectar secretion phenotypes downstream of JA.

### The JA Receptor COI1 Is Not Required for Nectar Secretion, but Is Required for the Nectary Auxin Response

**FIGURE 4:**
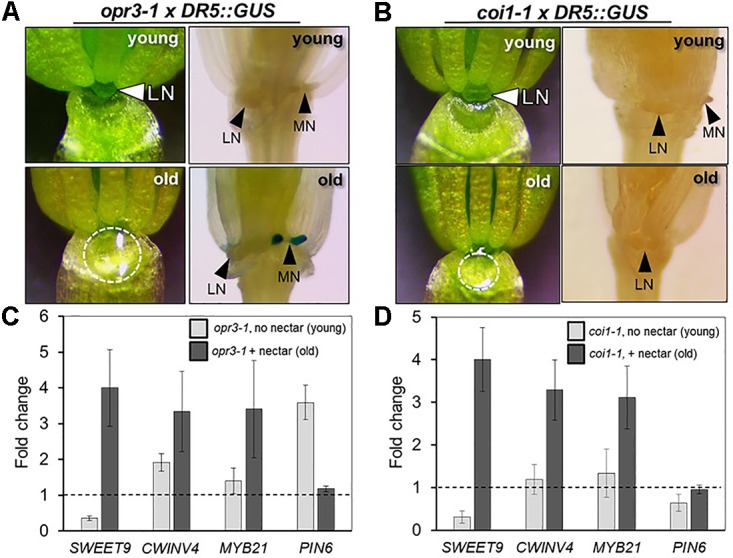
The JA receptor COI1 is not required for nectar secretion, but is required for the nectary auxin response. The ‘young’ flowers of *opr3-1* **(A)** and *coi1-1* **(B)** do not produce nectar, whereas the corresponding ‘old’ flowers secrete nectar. ‘Old’ *opr3-1* flowers (with nectar) display a strong auxin response in nectaries (**A**, lower right), but the corresponding ‘old’ *coi1-1* flowers with nectar do not (**B**, lower right). The expression of key genes involved in nectar secretion was examined in whole flowers by qRT-PCR in both ‘young’ (no nectar) and ‘old’ (with nectar) of *opr3-1* **(C)** and *coi1-1* **(D)**. qRT data was normalized to Col-0 (dashed line).

In light of the apparent dependence of nectar secretion on JA, an interesting phenotype was observed in the ‘old’ flowers of the JA-biosynthesis mutants *dad1-1* and *opr3-1*. Specifically, ‘young’ *dad1-1* and *opr3-1* flowers behaved similarly to those of the JA-deficient *aos-2* by not producing nectar, but their ‘old’ flowers secreted large nectar droplets (**Supplementary Figure S2B** and **Figure 4A**), unlike older *aos-2* flowers (**Figure 3B**). However, these results should be considered in the context of the fact that neither *dad1-1* nor *opr3-1* are completely JA-deficient [there is partial functional redundancy in the case of *DAD1* (Ishiguro et al., 2001)] and *opr3-1* is a leaky mutant (Chehab et al., 2011). Regardless of this fact, ‘young’ nectarless flowers crossed with DR5::GUS displayed no nectary auxin response, whereas the older flowers with nectar did have a robust auxin response specifically in the nectaries, although only in the median nectaries in the case of *opr3-1* (**Figure 4A** and **Supplementary Figure S2B**), again reinforcing a connection between auxin signaling and nectar secretion downstream of JA.

Previous reports have shown that the F-box protein CORONATINE INSENSITIVE 1 (COI1), which is a component of a JA receptor critical for JA-mediated signaling, is indispensable for floral starch metabolism and nectar secretion in tobacco flowers (Wang et al., 2014). Therefore, in addition to our JA biosynthesis mutants, we examined the reportedly JA-insensitive *coi1-1* in Arabidopsis. As also observed in *aos-2* × SWEET9pro::*iaa*M, *dad1-1* and *opr3-1*, there was a distinct difference in floral nectar secretion between ‘young’ and ‘old’ flowers. ‘Young’ *coi1-1* flowers (equivalent to Stage 14 in Col-0), showed a nectar-less phenotype, whereas the ‘old’ flowers did produce nectar (**Figure 4B**), suggesting the presence of a COI1-independent pathway responsible for regulating certain aspects of nectar secretion in Arabidopsis. Further support for the notion of a COI1-dispensible route for nectar secretion comes from the fact that both exogenous NAA and MeJA induced nectar secretion in ‘young’ *coi1-1* flowers (**Supplementary Figure S4**).

We also examined a mutant of *JASMONATE RESISTANT 1 (JAR1)* (AT2G46370), *jar1-11*, which is deficient in the JAR1 enzyme that conjugates JA to isoleucine in order to generate the bioactive JA-Ile. JA-Ile interacts directly with COI1 to mediate JA signal transduction (Staswick and Tiryaki, 2004). Interestingly *jar1-11*, which has previously been shown to accumulate JA-Ile levels to only 10% of that in WT upon wounding (Suza and Staswick, 2008), still produced nectar (**Supplementary Figure S5**) suggesting that either the low levels of JA-Ile are adequate for signaling nectar production or that there may be an alternative signaling module involved for nectar secretion.

There were notable changes in the expression of essential genes required for nectar production in the *aos-2* mutant that may account for the absence of nectar (**Figures 2**, **3**). We thus hypothesized that a similar pattern of gene expression may be observed in *opr3-1* and *coi1-1* that may cause the ‘young’ and ‘old’ nectar phenotype. Young nectarless *opr3-1* and *coi1-1* flowers showed a strong reduction in the expression of *SWEET9* (required for nectar production), whereas older flowers with nectar displayed a ∼3-to-4-fold induction of *SWEET9*, *CWINV4*, and *MYB21* relative to wild-type) (**Figures 4C,D**). Interestingly, *PIN6* expression begins 3.5-fold higher in ‘young’ *opr3-1* flowers but drops off to wild-type levels in ‘old’ flowers (**Figure 4C**). No significant difference in *PIN6* (auxin transporter) expression was observed in either the ‘young’ or ‘old’ flowers of *coi1-1* relative to Col-0 (**Figure 4D**). However, *coi1-1* × DR5::GUS failed to display an auxin response in either ‘young’ (no nectar) or ‘old’ (with nectar) flowers (**Figure 4B**). These results imply the presence of a COI1-independent pathway for nectar secretion, but that COI1 is required for the auxin responses observed in the nectaries of other JA-biosynthesis mutants.

### JA Responses Are High in Nectaries During Nectar Production as Revealed by a Biosensor

A recently developed JA reporter system can be used to observe jasmonate signaling *in planta* (Larrieu et al., 2015). In brief, this system was created based on the knowledge that the Jas motif in JAZ proteins is required for their degradation upon JA-induced signaling, thus releasing the repression of JA-inducible genes (Yan et al., 2009; Wasternack and Hause, 2013). The Jas motif of AtJAZ9 was fused with VENUS, a fast maturing YFP and placed under the control of the CaMV 35S promoter. These lines were designated as Jas9-VENUS and robust studies confirmed their use as a JA biosensor (Larrieu et al., 2015). A mutant version, mJas9-VENUS, was also engineered such that it cannot be targeted for degradation by COI1, even in the presence of bioactive JA-Ile (Larrieu et al., 2015). Thus, Jas9-VENUS is rapidly degraded in tissues undergoing active JA responses and an be monitored by a reduction in fluorescence, whereas stabilized mJas9-VENUS is not degraded.

**FIGURE 5:**
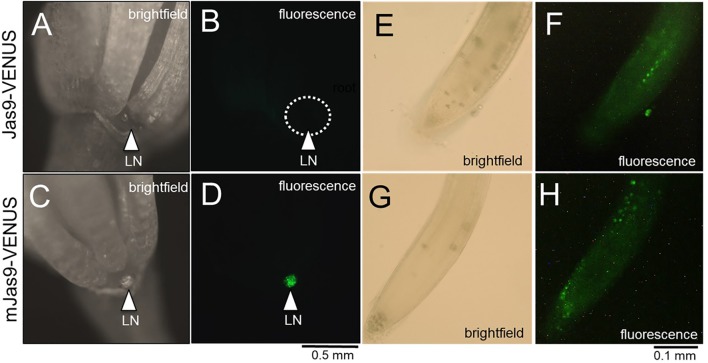
The JA response in mature lateral nectaries. Mature lateral nectaries (Stage 14 flowers with nectar) were evaluated for active JA responses via the Jas9-VENUS and mJas9-VENUS system. Jas9-VENUS is degraded in the presence of active JA signaling (decreased fluorescence), whereas mJas9-VENUS is not degraded (stable fluorescence). **(A)** Jas9::VENUS brightfield and **(B)** corresponding fluorescence in a Stage 14 nectary. **(C)** mJas9::VENUS brightfield and **(D)** corresponding fluorescence image in a Stage 14 nectary. The roots of both Jas9::VENUS **(E,F)** and mJas9::VENUS **(G,H)** display strong VENUS-dependent signal (punctate spots represent nuclei in **G,H**).

We hypothesized that the JA signaling and response in the nectaries of a Stage 14 flower would be high and thus lead to active degradation of Jas9-VENUS, manifesting as no or very low signal in a Jas9-VENUS line whereas the mJas9-VENUS line would show a nuclear signal in the nectaries. To confirm that the plants being tested indeed had a VENUS-dependent fluorescence signal, 5-day-old seedlings grown on half strength MS media were imaged with a fluorescence microscope. Seedlings with a positive signal for both Jas9- and mJas9-VENUS signal in the roots (**Figures 5E–H**) were transplanted to soil. Fluorescence imaging of Stage 14 flowers reveal a strong signal in the nuclei of the nectaries of the mJas9-VENUS lines whereas the signal was absent in the Jas9-VENUS nectaries (**Figures 5A–D**) suggesting that JA signaling was active in nectaries during nectar production. To confirm that the signal was not due to autofluorescence we imaged Stage 14 mJas9-VENUS flower nectaries with a confocal microscope and spectrally unmixed the image to differentiate VENUS from autofluorescence (**Supplementary Figure S6**). These results indicate that JA is acting as primary signal directly in the nectaries and not in other proximal floral tissues that may be transmitting secondary signals to the nectaries.

### *MYB21* Is Required for Nectar Production

The transcription factor MYB21 is JA inducible and has been shown to play a critical role in stamen maturation and overall flower development (Stintzi and Browse, 2000; Stracke et al., 2001; Mandaokar et al., 2006; Cheng et al., 2009; Song et al., 2011). More specifically to nectar regulation, MYB305, the tobacco ortholog of MYB21, was reported to directly play a role in nectar production through the regulation of starch metabolism in tobacco flowers (Liu and Thornburg, 2012; Wang et al., 2014). *MYB305* mutants accumulate lower levels of starch in their nectaries and have reduced levels of nectar production (Liu and Thornburg, 2012). Reeves et al. (2012) also showed that 20 nectary specific genes were down-regulated in the *myb21-5 myb24-5* double mutant. Even with these reports in the literature, there has yet to be a report on the role of *MYB21* in Arabidopsis nectary function.

**FIGURE 6:**
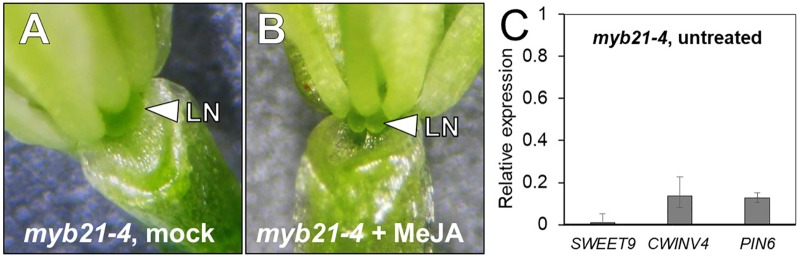
*MYB21* is required for nectar production. The flowers of mock- and MeJA treated *myb21-4* do not produce nectar **(A,B)**. The expression of key genes involved in nectar secretion was examined in whole untreated *myb21-4* flowers by qRT PCR **(C)**. Fold-change was calculated relative to Col-0 (wild-type expression = 1).

*myb21-4* is a null mutant previously described as having a premature stop codon (Trp116^∗^) (Reeves et al., 2012). As might be expected, the flowers of these mutants produce no nectar (**Figure 6A**). Perhaps unsurprisingly, nectar production was not rescued by exogenous application of MeJA (**Figure 6B**) since MYB21 is required downstream of JA signaling (Reeves et al., 2012). To further elucidate what genes MYB21 may be regulating we examined the expression of *SWEET9*, *CWINV4*, and *PIN6* via qRT-PCR analysis. All three genes showed a severe reduction in expression (>80%) (**Figure 6C**). Our data supports that the activity of MYB21 in the floral nectary indeed works downstream of JA and has a critical role in regulating the expression of several genes known to be essential for nectar production.

### Starch Metabolism Is Altered in Nectaries of JA Mutants

**FIGURE 7:**
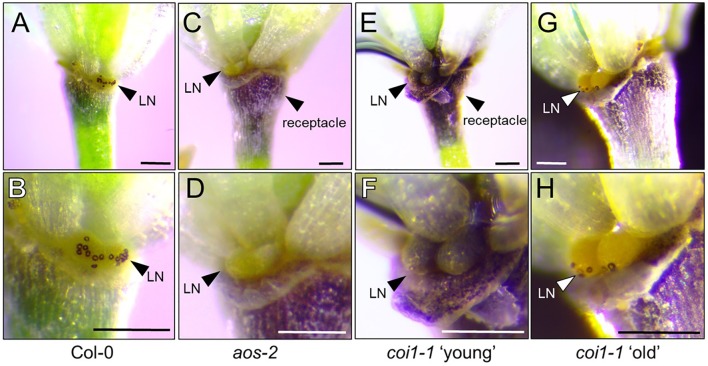
Starch accumulation patterns in *aos-2* and *coi1-1* nectaries. Each pair of images are of the same flower at different magnifications stained for starch accumulation. **(A,B)** Wild-type Col-0; **(C,D)** *aos-2*; **(E,F)** *coi1-1* ‘young’, **(G,H)** *coi1-1* old. Arrowheads either point out the lateral nectaries (LN) or the receptacle. Note the stained stomates in **(B,H)**. Scale bars = 100 microns.

Starch metabolism has been shown to be important in the process of proper nectary function (Paschold et al., 2008; Ruhlmann et al., 2010; Lin et al., 2014; Wang et al., 2014). Starch buildup is required in nectaries prior to nectar production and is rapidly broken down to produce sucrose and hexoses during the secretory phase. In tobacco, starch metabolism was severely compromised in a *coi1* mutant leading to an absence of nectar production (Wang et al., 2014). We hypothesized that *aos-2*, a JA biosynthetic mutant, would also display defects in starch metabolism. To test this hypothesis, Stage 14 WT and *aos-2* flowers were stained with Lugol’s iodine and imaged under a dissecting microscope. The distinct staining of the flower peduncle with iodine suggests a buildup of starch whereas lesser staining is consistent with the breakdown of the starch for nectar production. Also, the stomata on the nectaries stain distinctively when nectar secretion is occurring. Our study correlates a breakdown of starch with nectar secretion in the WT flowers (**Figures 7A,B**) whereas the *aos-2* flower peduncles have increased starch accumulation and a lack of stomatal staining (**Figures 7C,D**), as expected by the nectarless phenotype. The starch staining patterns observed in *aos-2* are very similar to those observed in *cwinv4* and *sweet9* mutants (Ruhlmann et al., 2010; Lin et al., 2014). Furthermore, since *coi1* mutants in tobacco showed defects in nectary starch metabolism and degradation (Wang et al., 2014), we hypothesized there may be starch defects in the Arabidopsis *coi1-1* mutant. In the nectarless ‘young’ flowers of *coi1-1* we indeed saw strong staining in both the peduncle and the nectary of the flower (**Figures 7E,F**), whereas in the ‘old’ flowers that do produce nectar, we see some breakdown of the starch in the peduncle and a mobilization of starch products to the guard cells of the nectary stomata (**Figures 7G,H**). This result confirms JA’s role in starch breakdown and subsequent nectar production.

## Discussion

### The Octadecanoic Acid Biosynthetic Pathway Is Required for Nectary Function

Arabidopsis mutants deficient in the octadecanoic pathway did not secrete nectar (**Figures 1–4**), particularly in ‘young’ open flowers that are the equivalent of Stage 14 wild-type flowers, which actively secrete nectar. Perhaps most telling, *aos-2* did not produce nectar droplets in either ‘young’ or ‘old’ flowers, although exogenous MeJA did restore nectar production (**Figures 1**, **2**). *AOS* is a single copy gene indispensable for the jasmonate biosynthesis pathway. Interestingly, the ‘old’ flowers of *dad1-1* and *opr3-1* did eventually produce nectar, which coincided with the expression of *SWEET9*, a sucrose transporter indispensable for nectar secretion (Lin et al., 2014). These results could possibly be explained by the fact that neither *dad1-1* nor *opr3-1* are completely JA deficient. *DAD1* encodes a phospholipase with extensive functional redundancy, as demonstrated by the fact that *dad1-1* mutants are defective in anther dehiscence and pollen development but are still able to accumulate JA upon wounding via the *DAD1* leaf homolog *DONGLE* (Ishiguro et al., 2001; Hyun et al., 2008). *OPR3* is reportedly essential for JA synthesis, but *opr3-1* has a leaky phenotype when exposed to pathogen attack (Chehab et al., 2011). It was also recently reported that in the absence of OPR3, OPDA could enter the β-oxidation route to produce 4,5-didehydro-JA which can subsequently act as a precursor to JA and JA-Ile (Chini et al., 2018). These results perhaps suggest that JA may still be able to accumulate to sufficient concentrations in *dad1-1* and *opr3-1* to restore the necessary signaling to rescue nectar secretion. However, it is important to note that anther dehiscence was not observed in any of the JA synthesis or response mutants, in either young or old flowers (data not shown). These results suggest that JA is not accumulating to high levels in these mutants and indicate that anther dehiscence is not required for nectar production.

### COI1 Is Dispensable for Nectar Secretion in Arabidopsis

The presence of nectar in ‘old’ *opr3-1* and *coi1-1* flowers (**Figure 4**) could also indicate the presence of an octadecanoic acid-dependent but JA- and COI1-dispensable signaling pathway in relation to nectary function. The substrate for OPR3 is *cis*-OPDA, which accumulates in *opr3-1* instead of being converted to JA (**Supplementary Figure S1**). *cis*-OPDA has been reported to be a potent signaling molecule that can regulate gene expression in either a COI1-dependent or COI-independent fashion (Dave and Graham, 2012). For JA responses to occur, JA-Ile is usually rapidly synthesized in response to an environmental or developmental cue which then binds the F-box protein COI1, which targets JAZ repressors for degradation via the 26S proteasome (Sheard et al., 2010; Pérez and Goossens, 2013; Wasternack and Hause, 2013). This subsequently liberates the transcription factors that drive JA-related gene expression. In the *jar1-11* mutant, where JA-Ile levels are severely compromised (Suza and Staswick, 2008), nectar production proceeds normally in ‘young’ flowers (**Supplementary Figure S5**). This suggests that the low levels of JA-Ile might suffice for nectar production or that nectar production might not require the well-established COI1-dependent JA regulation pathway in Arabidopsis. This latter supposition further gains support from the observation that nectar production proceeds normally in older flowers of the *coi1-1* loss-of-function mutant. It is remarkable though that younger *coi1-1* flowers produced no nectar even at Stages 13–14 when nectar secretion normally begins, indicating a COI1-dispensible pathway might require a time lag in responding to OPDA- or JA-dependent signaling. This delayed nectar phenotype is perhaps even more interesting in light of the reports that JA-Ile/COI1 signaling in wild tobacco flowers (*Nicotiana attenuata*) is required for nectar production (Stitz et al., 2014). Overall, this discrepancy should be further explored across species to better understand the conserved processes that are essential for nectar regulation.

### *MYB21* Is the Apparent Ortholog of Tobacco *MYB305*

**FIGURE 8:**
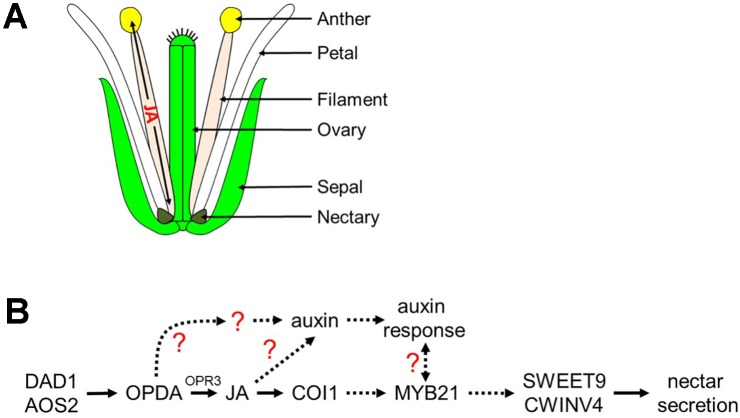
Proposed model of jasmonic acid migration after synthesis in the filament and regulation of nectar secretion. **(A)** JA synthesized in the anther filament migrates down the filament to the nectary to promote nectar secretion, and travels to the anther where it promotes the dehiscence of anthers and pollen maturation. **(B)** JA regulation of nectar secretion. Under standard conditions, JA or its precursors are synthesized in the filament leading to the induction of *MYB21* expression which in turn is required for nectar secretion via the expression of *SWEET9* and *CWINV4*. JA also regulates auxin responses within the nectaries, another hormonal factor involved in nectar secretion. A possible role for a JA- and COI1-dispensible pathway via OPDA for nectar secretion is indicated; dashed arrows represent hypothetical interactions based on limited data.

*MYB21* is a JA-responsive transcription factor previously reported to be important for stamen elongation and floral maturation (Reeves et al., 2012). It is also closely related to tobacco *MYB305* (*NtMYB305*) (Ren et al., 2007), which is required for nectar production and the expression of nectary-specific genes (Liu et al., 2009; Liu and Thornburg, 2012). In our study, *MYB21* expression was enhanced in *aos-2* flowers exogenously treated with MeJA, supporting its JA-inducibility (**Figure 2**). *myb21-4* flowers also did not produce nectar and also lacked the expression of genes required for nectar production – AtSWEET9, AtCWINV4, and AtPIN6 all displayed large decreases in expression in *myb21-4* (**Figure 6**). SWEET9 acts as a sucrose transporter, putatively transporting sucrose out of nectary parenchyma cells (Lin et al., 2014). After sucrose export, CWINV4 converts this disaccharide into its hexose monomers, glucose and fructose (Ruhlmann et al., 2010). Null mutants for these genes phenocopy one another as neither produce nectar, while maintaining normal nectary structure, and have heavy starch accumulation in the floral receptacle (Ruhlmann et al., 2010; Lin et al., 2014). The regulation of these nectar sugar transporters and invertases is vital for proper nectar production and secretion. Thus, MYB21 appears to regulate both *CWINV4* and *SWEET9* expression, in addition to *PIN6*, but it is currently unknown if MYB21 directly or indirectly controls their expression. It was interesting to note the expression of *SWEET9* was strongly reduced in the younger flowers of JA mutants such as *aos-2*, *opr3-1*, and *coi1-1* but gradually increased in the older flowers or in response to endogenous and exogenous auxin. These results suggest that SWEET9 expression proceeds via a COI1-dispensable, but MYB21-dependent route (**Figure 8**).

### Crosstalk Between JA and Auxin Pathways

There are a number of well-known interactions between auxin (IAA) and JA in plants, both in terms of homeostasis and downstream response (Pérez and Goossens, 2013; Wasternack and Hause, 2013). One of the key discoveries of our study was a further elucidation for the role of JA during nectar production and how it coordinates with auxin responses. Our study reveals that treatment of flowers with exogenous synthetic auxin (1-NAA) can rescue nectar production defects in JA biosynthetic mutants such as *aos-2* and *dad1-1*, which fail to produce nectar in ‘young’ open flowers (**Figure 2**). We also found that auxin responses in the nectaries of the ‘young’ open flowers of *dad1-1, aos-2* and *opr3-1* were absent as revealed by the DR5::GUS auxin reporter system. This suggests that the octadecanoic acid biosynthetic pathway is required for nectary auxin responses and subsequent nectar production. Auxin responses reappeared in *aos-2* flowers treated with MeJA suggesting that jasmonates can increase auxin biosynthesis in the nectaries. It remains to be tested whether free auxin levels are indeed diminished in the mutant flowers and whether they do increase after the MeJA treatment. However, we did demonstrate that nectary-derived auxin biosynthesis (via SWEET9pro::*iaa*M) can rescue nectar production in the *aos-2* background, likely by inducing SWEET9 expression (**Figure 3**). Cumulatively, these results indicate that auxin acts downstream of JA to induce nectar secretion.

The complete absence of an auxin response in *coi1-1* nectaries, even after nectar production occurred in older flowers, is particularly interesting (**Figure 4**). The possibility that nectar production occurs independently of COI1 in Arabidopsis has been discussed previously. This suggests that nectar production and a canonical auxin response via the TIR1 pathway in the nectary are separable events. Both auxin and JA use a similar mechanism of signaling by forming specialized co-receptor complexes known as SKP1-Cullin-F-box protein (SCF) E3 ubiquitin ligase complexes. TIR1 and COI1 act as the F-box proteins providing specificity for auxin and JA in these complexes, respectively. This complex subsequently binds their target proteins, Aux/IAA (auxin) and JAZ (JA), respectively, which are then degraded via the ubiquitin-proteasome system (Kelley and Estelle, 2012) thus allowing hormone specific transcriptional activation to proceed. Both these hormones are perceived by shared components of a SCF-E3 ligase system, and it has been demonstrated that disruption in the complexes can cause an impairment of hormonal responses (del Pozo et al., 2002; Quint et al., 2005; Moon et al., 2007; Pérez and Goossens, 2013). Thus, there is a possibility that the loss of COI1 disrupts the equilibrium of the shared aspects of the ubiquitin-proteasome system thus affecting auxin signaling and responses too.

### An Alternative Mechanism of JA Involvement in Nectar Production?

We should also consider alternative routes of how jasmonates and related oxylipins might affect nectar production. A potential for JA regulation of nectar secretion via control of water movement also exists. Proper timing of pollen release and nectar secretion in flowering plants can be vital for reproduction, therefore, there may be a close singular upstream mechanism between anther dehiscence and nectar secretion. Baum et al. (2001) characterized many male sterile mutants to be deficient in nectar production. This same correlation between anther dehiscence/male-sterility and nectar production was observed in different male sterile mutants of our study. Ishiguro et al. (2001) determined that water transport was halted in the vascular tissue of *dad1-1* anthers and suggested that JA regulates water transport in the male organs. Later, Ruhlmann et al. (2010) suggested that the high amount of nectar sugars leads to the flow of water out of the nectary, creating the nectar droplet presented at the base of the sepal. Perhaps water transport in the nectaries is disrupted by the lack of JA which leads to the absence of nectar in the JA synthesis and response mutants. Therefore, only when the proper JA response can be restored in the JA synthesis mutants can the water transport/nectar secretion be restored as well. However, the molecular mechanism by which water flow is disrupted to *dad1-1* stamens, and perhaps nectaries, is currently unknown.

Cumulatively, the results from this study implicate the octadecanoic acid biosynthetic pathway and auxin as indispensable regulators of nectar secretion independent of *COI1* in Arabidopsis. We propose a model (**Figure 8**) in which *cis*-OPDA may act as a signaling molecule upstream of *MYB21* and auxin responses leading to the expression of *SWEET9* and *CWINV4*. Still, certain aspects of the proposed model are unclear, particularly the connection between the auxin response and *MYB21* (dashed line with question mark), as exogenous treatment of *aos-2* flowers with NAA induced *SWEET9* expression and nectar secretion while concurrently downregulating *MYB21* expression (**Figure 2**). A possible explanation for the apparent discrepancy between *MYB21* and *SWEET9* expression in auxin-treated *aos-2* flowers is that qRT-PCR analyses were done with whole flowers (due to the extremely small size of Arabidopsis nectaries) and *MYB21* is not a nectary-specific gene (Reeves et al., 2012), unlike *SWEET9* and *CWINV4* (Ruhlmann et al., 2010; Lin et al., 2014). Therefore, it is possible that *MYB21* expression might be downregulated in non-nectary tissues of NAA-treated *aos-2* flowers, while actually being upregulated in nectaries. Further studies will focus on examining this crosstalk and IAA, JA, and *MYB21* in nectar secretion, as well as their respective control of *CWINV4* and *SWEET9* expression.

## Author Contributions

AS, RR, PK, MJ, and CC designed and executed the experiments. AS, RR and CC wrote the final manuscript.

## Conflict of Interest Statement

The authors declare that the research was conducted in the absence of any commercial or financial relationships that could be construed as a potential conflict of interest.
