# Supplementary material for: The Octadecanoid Pathway, but Not COI1, Is Required for Nectar Secretion in Arabidopsis thaliana
Source: Front Plant Sci. 2018 Aug 8;9:1060. doi: 10.3389/fpls.2018.01060 (PMC6092685; doi:10.3389/fpls.2018.01060)

Supplementary Material

The octadecanoid pathway, but not COI1, is required for nectar secretion in Arabidopsis thaliana

Anthony Schmitt^1^, Rahul Roy^1^, Peter Klinkenberg^1^, Mengyuan Jia1^Ɨ^, Clay Carter^1*^

^1^Department of Plant and Microbial Biology, University of Minnesota Twin Cities, St. Paul, MN, USA, ^Ɨ^Present address: Huck Institute of Life Sciences, Pennsylvania State University, University Park, PA, USA

*** Correspondence:** Dr. Clay Carter: [cjcarter@umn.edu](mailto:cjcarter@umn.edu)

##
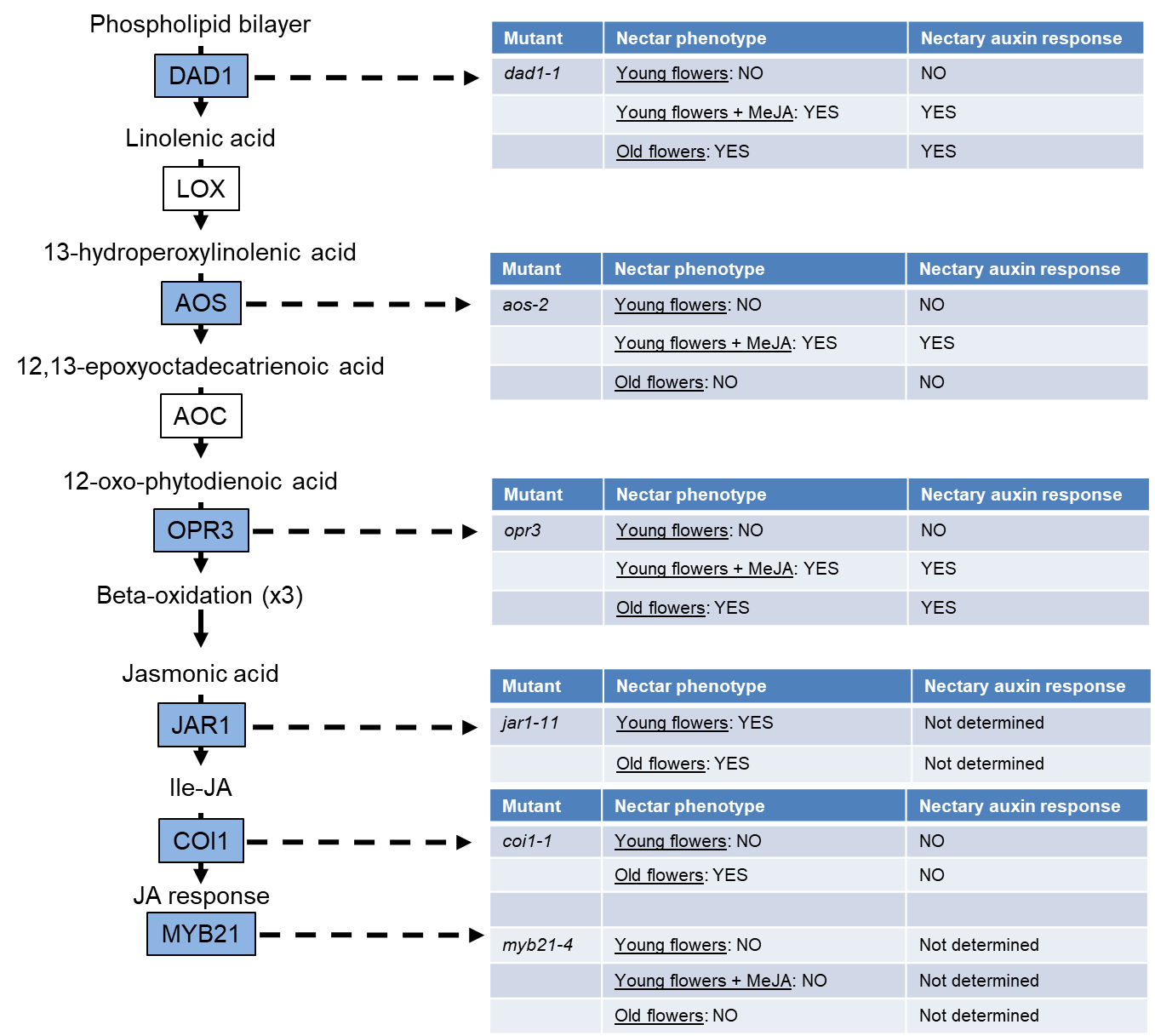
Supplementary Figures

**Supplementary Figure 1.** Summary of octadecanoic biosynthetic pathway and mutant phenotypes in relation to nectar/y phenotypes

**
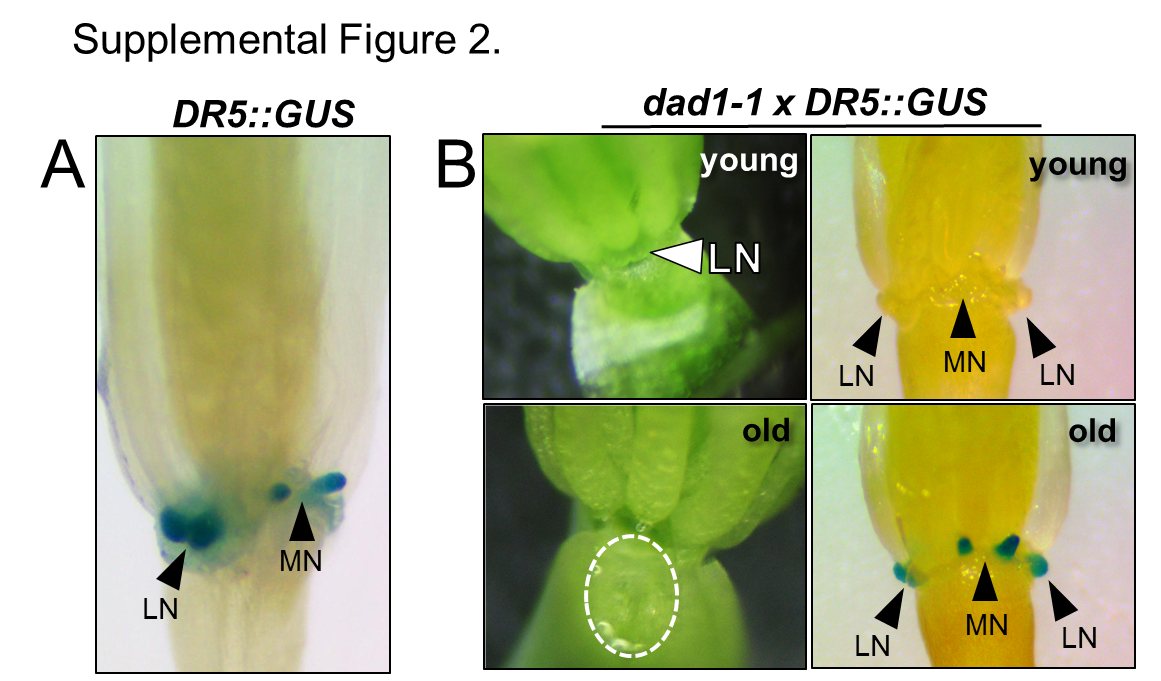
**

**Supplementary Figure 2.** (A) A wild-type plant carrying the auxin-responsive DR5::GUS reporter displays a strong auxin response in the nectaries of Stage 14 (fully open) flower. (B) ‘Young’ *dad1-1* flowers do not secrete nectar or display a nectary auxin response (upper panels), whereas ‘old’ ones do (lower panels).


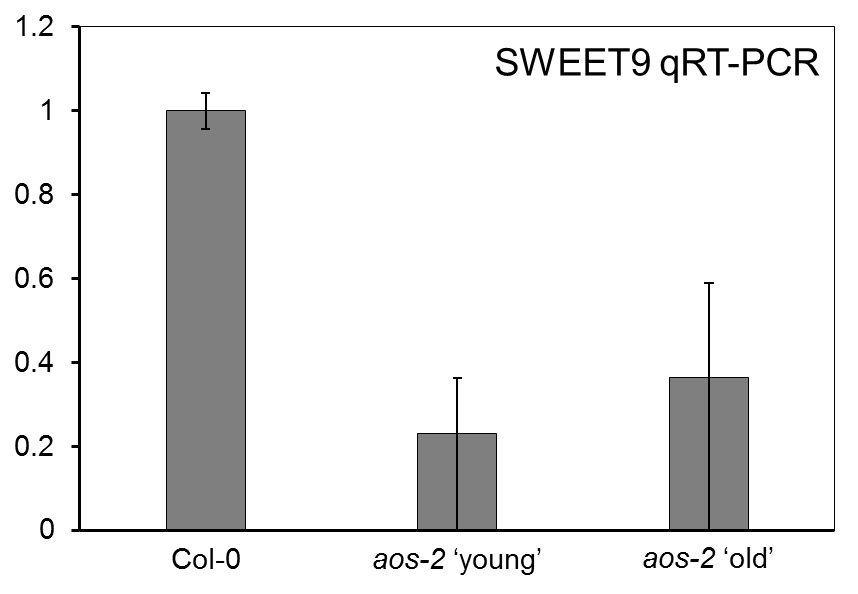


**Supplementary Figure 3. Relative *SWEET9* transcript abundance in wild-type and ‘young’ and ‘old’ *aos-2* flowers as determined by qRT-PCR.** Note that *SWEET9* is down-regulated in both *aos-2* ‘young’ and ‘old’ flowers relative to wild-type (Col-0) flowers at Stage 14 (actively secreting nectar, but before dehiscence; equivalent to ‘young’ *aos-2* flowers).

**
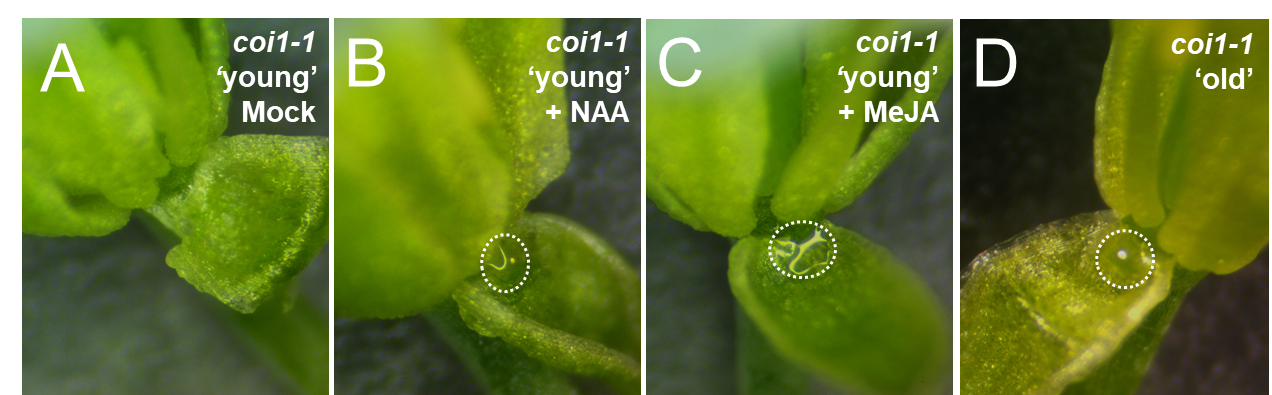
Supplementary Figure 4. Exogenous auxin and methyl jasmonate induce nectar secretion in ‘young’ *coi1-1* flowers.** Mock treated ‘young’ *coi1-1* flowers (A) do not produce nectar, whereas ones treated with 10 μM NAA (B) or 500 μM methyl jasmonate (C) both produce nectar. ‘Old’ *coi1-1* flowers (D) naturally produce nectar, as described in Fig. 4B. Dashed circles outline the presence of nectar droplets. These results suggest the presence of a COI1-dispensible route for nectar secretion in Arabidopsis.


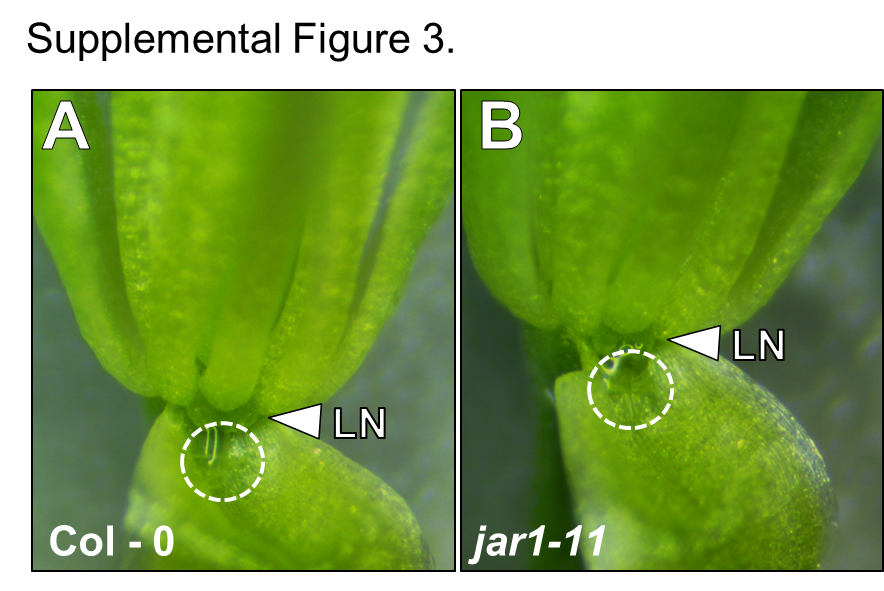


**Supplementary Figure 5. Mutants deficient in jasmonyl-isoleucine can still produce nectar.** Both wild-type Col-0 (A) and a mutant, *jar1-11* (B), deficient in the bioactive form of JA, jasmonyl-isoleucine, produce nectar. Arrowheads indicate the location of the lateral nectaries (LN) and dashed circles outline the presence of nectar droplets.


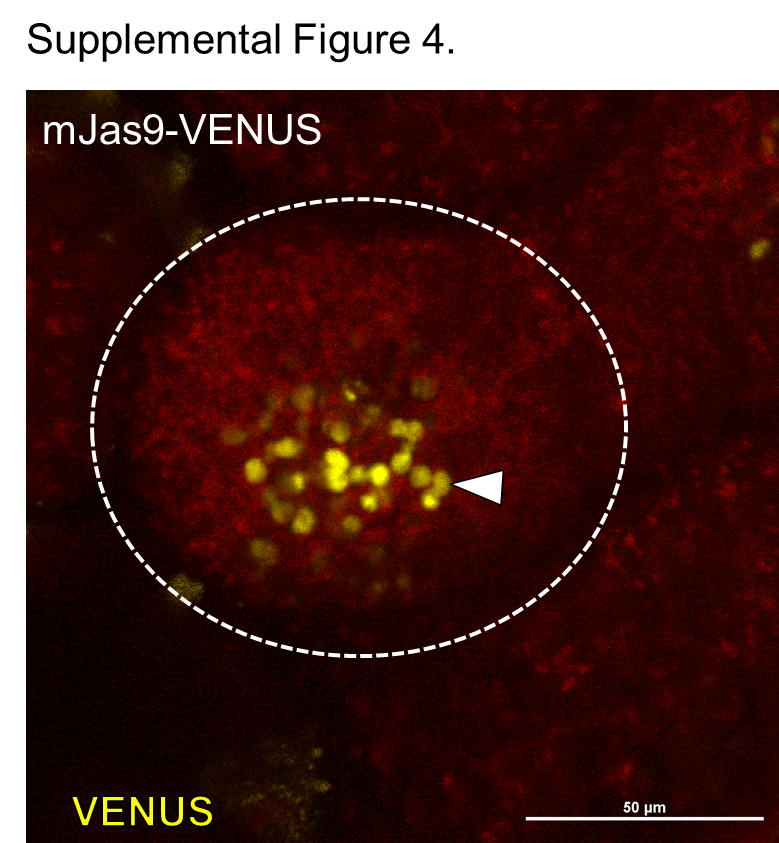


**Supplementary Figure 6. VENUS signal in lateral nectaries of reporter lines is not due to autofluorescence.** Spectrally unmixed confocal image of a Stage 14 flowers of the mJas9-Venus reporter line differentiating autofluorescence from the emission derived from VENUS, which seems to be in the nuclei (arrowhead)

## Supplementary Tables

List of oligos used in the study


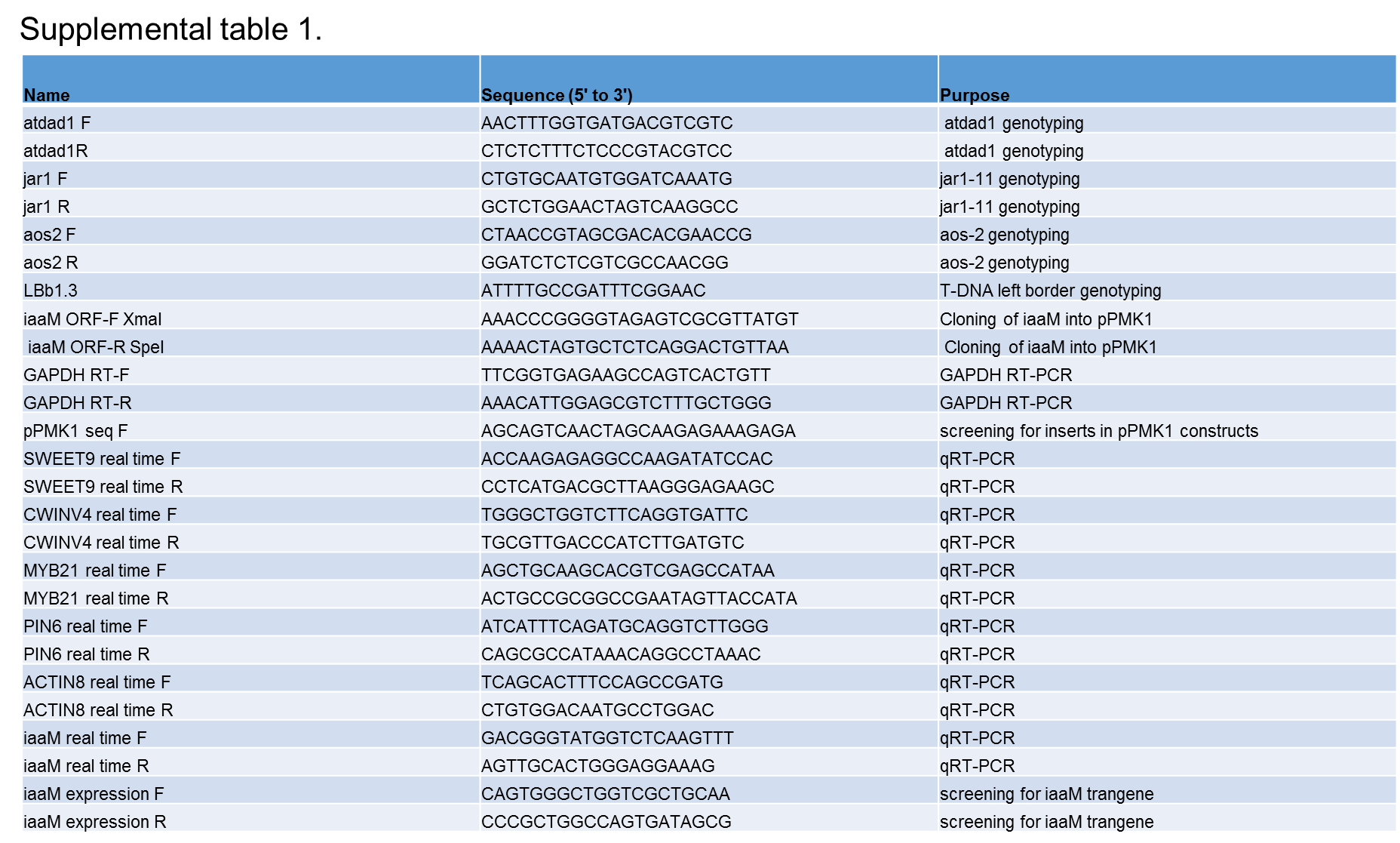

Supplement: Supplementary file 1 [file Data_Sheet_1.docx]
